# Association between Statin Use and Meniere’s Disease: Results from a National Health Screening Cohort

**DOI:** 10.3390/ijerph18168692

**Published:** 2021-08-17

**Authors:** So Young Kim, Dae Myoung Yoo, Chanyang Min, Hyo Geun Choi

**Affiliations:** 1 Department of Otorhinolaryngology-Head & Neck Surgery, CHA Bundang Medical Center, CHA University, Seongnam 13496, Korea; sossi81@hanmail.net; 2 Hallym Data Science Laboratory, Hallym University College of Medicine, Anyang 14068, Korea; ydm1285@naver.com (D.M.Y.); joicemin@naver.com (C.M.); 3 Graduate School of Public Health, Seoul National University, Seoul 08826, Korea; 4 Department of Otorhinolaryngology-Head & Neck Surgery, Hallym University College of Medicine, Anyang 14068, Korea

**Keywords:** Meniere’s disease, hydroxymethylglutaryl-CoA reductase inhibitors, cohort studies, case-control studies

## Abstract

The protective effects of statins against inner ear diseases have been suggested. This study investigated the relationship between previous statin use and the occurrence of Meniere’s disease (MD). Participants ≥40 years old in the Korean National Health Insurance Service-Health Screening Cohort 2002–2015 were enrolled. A total of 7734 MD participants were matched with 38,670 comparison participants. The dates of statin prescriptions for the 2 years before the onset of MD were examined. A conditional logistic regression analysis was performed to estimate the odds ratios (ORs) of statin use for MD. Regarding the different types of statins, lipophilic statins, but not hydrophilic statins, were associated with lower odds of MD in the <65 year-old group (adjusted OR = 0.81, 95% CI = 0.68–0.97, *p* = 0.023). Prior statin use did not show association with MD in the adult population. Regarding the different types of statins, lipophilic statin use was related to a lower rate of MD in a middle-aged population.

## 1. Introduction

Statins are lipid-lowering agents that inhibit HMG-CoA reductase [1]. They are widely used for the treatment and prevention of dyslipidaemia and other cardiovascular disorders [2]. A number of randomized clinical trials demonstrated decreases in all-cause (risk ratio [RR] = 0.86, 95% confidence intervals [95% CI] = 0.80–0.93) and cardiovascular (RR = 0.69, 95% CI = 0.54–0.88) mortality related to statin therapy [2]. In addition, a growing number of reports have widened the indications of statin prescription for inflammatory diseases, such as asthma and chronic obstructive pulmonary disease, and neurologic disorders, such as Alzheimer’s disease and Parkinson’s disease [3]. The multiple pharmacologic actions of statins support the broad indications of statins [4]. Statins have been suggested to have anti-inflammatory and antioxidative effects as well as lipid-lowering effects [4]. On the other hand, the potential adverse effects of statins have been proposed for muscle weakness, memory problems, and sudden sensorineural hearing loss [5,6].

Meniere’s disease (MD) is an inner ear abnormality that is related to endolymphatic hydrops [7,8,9]. Patients with MD suffer from recurrent symptoms of vertigo, hearing loss, and tinnitus [10,11]. The prevalence of MD is estimated to be approximately 50–200 per 100,000 adults, and this disease is most common in the middle-aged population [12]. Although most patients with MD share the common pathology of endolymphatic hydrops, the aetiology of hydrops could be multifactorial and include primary endolymphatic hydrops and secondary endolymphatic hydrops due to autoimmune inner ear disease [13]. In addition, the presence of endolymphatic hydrops is not sufficient for the occurrence of MD [14]. Thus, other etiologies may contribute to MD [15,16]. Other possible pathologies have been suggested such as autonomic nervous system, parasympathetic defects, sympathetic defects, migraine, and allergies [11,17,18,19,20]. A few studies have suggested that vascular insufficiency may be associated with MD [21]. Among the patients diagnosed with definitive MD, as many as 65.6% (21/32) of patients showed abnormal findings in cerebrospinal venous flow, which was higher than the percentage of healthy comparison participants (25%, *p* < 0.001) [21].

Because vascular disorders and immune disease were suggested to cause MD, it can be hypothesized that the alleviation of these problems using statins may protect against the occurrence of MD. To test this hypothesis, the duration of previous statin use was compared between the MD and comparison groups. Because responses to statins were reported to be different according to age and sex, subgroup analyses were conducted according to age and sex. In addition, the types of statins, namely, hydrophilic and lipophilic statins, were reported to exhibit differences in therapeutic efficacy in cardiovascular diseases [22]. Thus, the relationship between statin use and MD was analyzed according to the types of statin. To the best of our knowledge, research on the association of statin use with MD is lacking.

## 2. Materials and Methods

### 2.1. Ethics

This study was approved by the ethics committee of Hallym University (2019-10-023). The Institutional Review Board exempted the requirement for written informed consent. We complied with the guidelines and regulations of the ethics committee of Hallym University for all analyses. This study complied with the Strengthening the Reporting of Observational Studies in Epidemiology guideline.

### 2.2. Study Population and Participant Selection

**Figure 1:**
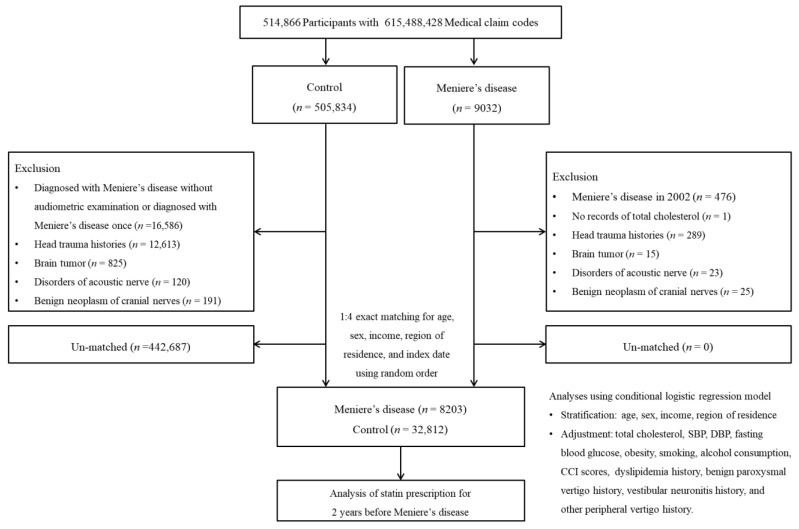
A schematic illustration of the participant selection process that was used in the present study. Of a total of 514,866 participants, 7734 Meniere’s disease participants were matched with 30,936 comparison participants for age, sex, economic level, and region of residence.

A detailed description of the Korean National Health Insurance Service-Health Screening Cohort data is provided elsewhere [23]. MD participants were selected from 514,866 participants with 615,488,428 medical claim codes from 2002 through 2015 (*n* = 9032). The comparison group was defined as not having MD from 2002 through 2015 (*n* = 505,834). To select participants who were diagnosed for the first time, MD participants diagnosed in 2002 and 2003 were excluded. MD participants who did not have records of total cholesterol levels were excluded (*n* = 1). The exclusion criteria for comparison participants were as follows: participants who were diagnosed with MD but did not undergo audiometric examination or were diagnosed with MD less than twice (*n* = 16,586), participants who were treated according to a history of head trauma (S00 to S09) ≥2 times with head and neck CT evaluations, and participants who were treated for brain tumors (C70 to C72) ≥2 times, disorders of acoustic nerves (ICD-10 code: H933) ≥2 times, and benign neoplasms of cranial nerves (D333) ≥2 times. Age, sex, economic level, and region of residence were matched between MD and comparison participants. To prevent bias when selecting the matched participants, the comparison participants were sorted using a random number order. The index date was defined as the date of diagnosis of MD and the index date of the matched MD participants for comparison participants. Participants who were deceased before the index date were excluded. During the matching procedure, 444,563 comparison participants were excluded. Finally, 7734 MD participants were matched 1:4 with 30,936 comparison participants (Figure 1).

### 2.3. Independent Variable

The prescription dates of statins were counted for 2 years before the index dates for both MD and comparison participants [24].

### 2.4. Dependent Variable

Participants who visited clinics due to MD (H810) ≥2 times, and who underwent audiometric examination (claim codes: E6931-E6937, F6341-F6348), both recorded in the Health Insurance Service, were enrolled [15].

### 2.5. Covariates

Five-year interval age groups were used as presented in Table 1. The lowest economic level group was designated as class 1, and the highest economic level group was classified as class 5 in economic level groups. Urban and rural areas were the categories for the region of residence [15]. Smoking status and the frequency of alcohol consumption were evaluated [15]. Obesity groups were classified using BMI (body mass index, kg/m^2^) [15]. The measurements of total cholesterol (mg/dL), systolic blood pressure (SBP, mmHg), diastolic blood pressure (DBP, mmHg), fasting blood glucose (mg/dL), and hemoglobin (g/dl) were classified based on the normal ranges [15].

Past medical histories were collected and counted as the Charlson Comorbidity Index (CCI) score [25]. A treatment history of ≥2 times was used as the diagnostic criteria for benign paroxysmal vertigo (ICD-10 code: H811), vestibular neuronitis (ICD-10 code: H812), other peripheral vertigo (ICD-10 code: H813), and dyslipidaemia (ICD-10 code: E78).

### 2.6. Statistical Analyses

Categorical variables were compared between the MD and comparison groups using the chi-square test. Continuous variables were compared using a Mann–Whitney U test.

The odds ratios (ORs) with 95% CIs of statin prescription (for 1 year) for MD were estimated with conditional logistic regression. In these analyses, crude model 1 (SBP, DBP, fasting blood glucose, total cholesterol, and dyslipidaemia history) and model 2 (model 1 plus obesity, smoking, alcohol consumption, CCI scores, benign paroxysmal vertigo history, vestibular neuronitis history, and other peripheral vertigo history) were used (Table 2). Additionally, statin prescriptions (for 1 year) were analyzed by dividing the patients according to hydrophilic statin and lipophilic statin use (Table 3).

For the subgroup analyses, we classified participants by age (<65 years old and ≥65 years old), sex (men and women), economic level (low and high), and region of residence (rural and urban).

We performed additional subgroup analyses using unconditional logistic regression. We divided participants by obesity, smoking, alcohol consumption, blood pressure, fasting blood glucose, total cholesterol, dyslipidaemia history, benign paroxysmal vertigo history, vestibular neuronitis history, and other peripheral vertigo history. Tests of interaction terms were conducted for all subgroups.

A *p*-value <0.05 was regarded as significant. Analyses were performed using SAS version 9.4 (SAS Institute Inc., Cary, NC, USA).

## 3. Results

The dates of statin use were different between the MD and comparison groups (50.77 [standard deviation = 112.8] days vs. 43.18 [107.0] days, *p* < 0.001, Table 1). The rates of alcohol consumption, CCI score, and dyslipidemia history were higher in the MD group than in the comparison group (*p* < 0.05).

The dates of statin use were associated with higher odds for MD in the crude model (OR = 1.26, 95% CI = 1.17–1.37, *p* < 0.001, Table 2). However, when adjusted for covariates, the dates of statin use were not associated with MD in model 1 or model 2 (adjusted OR [aOR] = 0.92, 95% CI = 0.83–1.02, *p* = 0.110). In the age and sex subgroups, all subgroups did not show an association of MD with the dates of statin use. Several additional subgroups including past smokers and current smokers and other peripheral vertigo showed lower odds for MD associated with statin use (Table S1). Other subgroups did not show an association between statin use and MD.

When statins were divided by type into lipophilic and hydrophilic statins, lipophilic statins were not related with MD in the overall population.

The <65-year-old group demonstrated low odds for MD with lipophilic statin use (aOR = 0.81, 95%CI = 0.68–0.97, *p* = 0.023). For hydrophilic statin use, there was no relationship of hydrophilic statin use with MD (Table 4).

## 4. Discussion

Previous statin use was not associated with MD in the overall adult population. However, the association of MD with statins was different according to the type of statin. Lipophilic statin use, but not hydrophilic statin use, was related to a low occurrence of MD in the middle-aged adult population. This study, for the first time, investigated the impact of statin use on MD. Although the underlying pathophysiology needs further research, the pleiotropic effects of statins on the inner ear and vascular system could be linked with a reduced occurrence of MD.

There has been no research on the impact of statins on the vestibular system. However, several previous studies suggested the possible protective effects of statins on the cochlea [26]. A few animal studies proposed that statin administration inhibited the oxidative stress response and inflammation of the cochlea [27]. A clinical trial showed a protective effect of atorvastatin against cisplatin-induced hearing loss [26]. Compared to patients without statin use, those with statin use demonstrated 0.47-fold lower odds of cisplatin-induced hearing loss (95% CI = 0.30–0.78) [26]. Systemic inflammatory responses have been reported in patients with MD [28]. Thus, the anti-inflammatory effects of statins could hinder the development of MD. Moreover, the beneficial effects of statins were proposed in noise-induced and idiopathic sudden sensorineural hearing loss as well as in cisplatin-induced and dyslipidaemia-induced hearing loss [29]. Therefore, statins could have multiple protective effects on inner ear disease.

The protective effects of statins on the vascular system could alleviate the vascular compromise of the inner ear, which could regulate the inner ear lymphatic circulation and prevent endolymphatic hydrops. Impaired labyrinthine microcirculation was suggested as one of the causes of endolymphatic hydrops [30]. The endolymphatic space has capillary microcirculation through the stria vascularis [30]. The increased hydrostatic pressure of the stria vascularis, or the occlusion of venous drainage through the vein of the vestibular aqueduct can disturb the endolymphatic flow, which induces endolymphatic hydrops [30]. A prospective case-comparison study reported a higher prevalence of chronic cerebrospinal venous insufficiency in patients with MD (80.1% [330/412] of MD patients vs. 11.8% [12/102] of comparison participants, *p* <0.001) [31]. In addition, MD patients who underwent vascular intervention showed greater improvements from aural fullness and hearing loss than those in the medication-only group [31]. Therefore, the improvement and protection of cardiovascular circulation through statin therapy may have beneficial effects on labyrinthine microcirculation in that it can suppress the occurrence of MD.

Lipophilic statin use was associated with a decreased risk of MD in this study. Lipophilic statins are lipid-soluble, thereby demonstrating a higher diffusion rate through lipid layers of cellular membranes [32]. Thus, the pharmacological effects of statins could be larger and longer with lipophilic statins than with hydrophilic statins. Although a meta-analysis study estimated a similar efficacy of hydrophilic and lipophilic statins on cardiovascular diseases [33], the protective effects of lipophilic statins were reported for certain diseases, such as hepatocellular carcinoma and hearing impairment [26]. According to age groups, the middle-aged population showed an inverse association of previous statin use with MD in the present study. Although many comorbidities were adjusted for in this study, the increased number of comorbidities in the elderly population could mitigate the protective effect of statins on MD. In addition, the high prevalence of MD in the middle-aged population might influence the solid association of prior statin use with MD.

This study used representative data from a large cohort. We randomly selected comparison participants who matched with study participants, thereby minimizing the potential selection bias. The various covariates of lifestyle factors, laboratory findings, and comorbidities, including other vestibular diseases, were adjusted to attenuate the confounding effects. However, because this study was based on health claim data, untreated or undiagnosed cases were not included. The results of vestibular function tests and audiometry tests were not available. Thus, the degree of hearing loss and vestibular dysfunction might be heterogeneous among MD patients. For statin use, this study collected data on statin prescriptions; however, compliance with statin prescriptions could not be checked. In addition, the statin dose prescribed did not account for the association of statin use with MD.

## 5. Conclusions

Previous statin use was not associated with the occurrence of MD in the adult population. However, in the middle-aged population, the inverse association of previous statin use with MD was presented with lipophilic statin use.

## Figures and Tables

**Table 1:** General characteristics of the participants.

Characteristics		Total Participants		
		Meniere’ Disease	Comparison	*p*-Value
Total number (*n*, %)		7734 (100.0)	30,936 (100.0)	
Age (years old) (*n*, %)	40–44	72 (0.9)	288 (0.9)	1.000
	45–49	470 (6.1)	1880 (6.1)	
	50–54	1130 (14.6)	4520 (14.6)	
	55–59	1336 (17.3)	5344 (17.3)	
	60–64	1257 (16.3)	5028 (16.3)	
	65–69	1231 (15.9)	4924 (15.9)	
	70–74	1115 (14.4)	4460 (14.4)	
	75–79	726 (9.4)	2904 (9.4)	
	80–84	318 (4.1)	1272 (4.1)	
	85+	79 (1.0)	316 (1.0)	
Sex (*n*, %)	Male	2752 (35.6)	11,008 (35.6)	1.000
	Female	4982 (64.4)	19,928 (64.4)	
Income (*n*, %)	1 (lowest)	1343 (17.4)	5372 (17.4)	1.000
	2	967 (12.5)	3868 (12.5)	
	3	1193 (15.4)	4772 (15.4)	
	4	1605 (20.8)	6420 (20.8)	
	5 (highest)	2626 (40.0)	10,504 (40.0)	
Region of residence (*n*, %)	Urban	3267 (42.2)	13,068 (42.2)	1.000
	Rural	4467 (57.8)	17,868 (57.8)	
Obesity (*n*, %) †	Underweight	152 (2.0)	809 (2.6)	<0.001 *
	Normal	2638 (34.1)	11,006 (35.6)	
	Overweight	2168 (28.0)	8337 (27.0)	
	Obese I	2541 (32.9)	9709 (31.4)	
	Obese II	235 (3.0)	1075 (3.5)	
Smoking status (*n*, %)	Nonsmoker	6249 (80.8)	24,360 (78.7)	<0.001 *
	Past smoker	812 (10.5)	3011 (9.7)	
	Current smoker	673 (8.7)	3565 (11.5)	
Alcohol consumption (*n*, %)	<1 time a week	5804 (75.0)	22,323 (72.2)	<0.001 *
	≥1 time a week	1930 (25.0)	8613 (27.8)	
Systolic blood pressure (*n*, %)	<120 mmHg	2387 (30.9)	9315 (30.1)	0.006 *
	120–139 mmHg	3806 (49.2)	14,948 (48.3)	
	≥140 mmHg	1541 (19.9)	6673 (21.6)	
Diastolic blood pressure (*n*, %)	<80 mmHg	3828 (49.5)	14,836 (48.0)	0.002 *
	80–89 mmHg	2749 (35.5)	40,998 (35.5)	
	≥90 mmHg	1157 (15.0)	5102 (16.5)	
Fasting blood glucose (*n*, %)	<100 mg/dL	4897 (63.3)	19,414 (62.8)	0.010 *
	100–125 mg/dL	2211 (28.6)	8678 (28.0)	
	≥126 mg/dL	626 (8.1)	2844 (9.2)	
Total cholesterol (*n*, %)	<200 mg/dL	3989 (51.6)	15,984 (51.7)	0.989
	200–239 mg/dL	2609 (33.7)	10,420 (33.7)	
	≥240 mg/dL	1136 (14.7)	4532 (14.6)	
CCI score (*n*, %)	0	4876 (63.0)	21,066 (68.1)	<0.001 *
	1	1412 (18.3)	4495 (14.5)	
	≥2	1446 (18.7)	5375 (17.4)	
Dyslipidemia history (*n*, %)		2949 (38.1)	9337 (30.2)	<0.001 *
Benign paroxysmal vertigo history (*n*, %)		1777 (23.0)	1161 (3.7)	<0.001 *
Vestibular neuronitis history (*n*, %)		506 (6.5)	288 (0.9)	<0.001 *
Other peripheral vertigo history (*n*, %)		1180 (15.3)	878 (2.8)	<0.001 *
The dates of statin prescription (days, mean, SD)		50.77 (112.8)	43.18 (107.0)	<0.001 *
The dates of hydrophilic statin prescription (days, mean, SD)		9.11 (51.38)	7.38 (46.73)	<0.001 *
The dates of lipophilic statin prescription (days, mean, SD)		41.66 (102.4)	35.78 (97.60)	<0.001 *

CCI, Charlson comorbidity index; SD, standard deviation, * Chi-square test or Mann–Whitney test. Significance at *p* < 0.05, †Obesity (BMI, body mass index, kg/m^2^) was categorized as < 18.5 (underweight), ≥ 18.5 to < 23 (normal), ≥ 23 to < 25 (overweight), ≥ 25 to < 30 (obese I), and ≥ 30 (obese II).

**Table 2:** Crude and adjusted odd ratios (95% confidence interval) of date of statin prescription (1 year) for Meniere’ disease with stratified subgroup according to age, sex, income, and region of residence.

Characteristics	Odds Ratios for Meniere’ Disease						*p* Value for Interaction
	Crude†	*p*-Value	Model 1 †‡	*p*-Value	Model 2 †§	*p*-Value	
Total participants (*n* = 38,670)							
Statin prescription (1 year)	1.26 (1.17–1.37)	<0.001 *	0.96 (0.88–1.06)	0.433	0.92 (0.83–1.02)	0.110	
Age < 65 years old (*n* = 21,325)							0.668
Statin prescription (1 year)	1.31 (1.15–1.49)	<0.001 *	0.90 (0.77–1.04)	0.167	0.85 (0.72–1.00)	0.051	
Age ≥ 65 years old (*n* = 17,345)							
Statin prescription (1 year)	1.24 (1.11–1.37)	<0.001 *	1.02 (0.90–1.15)	0.740	0.98 (0.86–1.13)	0.84	
Men (*n* = 13,760)							0.675
Statin prescription (1 year)	1.26 (1.09–1.45)	0.002 *	0.92 (0.79–1.09)	0.347	0.88 (0.74–1.05)	0.158	
Women (*n* = 24,910)							
Statin prescription (1 year)	1.27 (1.15–1.40)	<0.001 *	0.98 (0.88–1.11)	0.803	0.94 (0.83–1.07)	0.343	
Low income (*n* = 17,515)							0.821
Statin prescription (1 year)	1.30 (1.15–1.47)	<0.001 *	0.93 (0.80–1.07)	0.319	0.88 (0.75–1.03)	0.117	
High income (*n* = 22,335)							
Statin prescription (1 year)	1.24 (1.11–1.38)	<0.001 *	0.99 (0.87–1.12)	0.876	0.95 (0.83–1.09)	0.462	
Urban residents (*n* = 16,335)							0.425
Statin prescription (1 year)	1.20 (1.06–1.36)	0.004 *	0.91 (0.79–1.05)	0.215	0.87 (0.74–1.02)	0.094	
Rural residents (*n* = 22,335)							
Statin prescription (1 year)	1.31 (1.18–1.46)	<0.001 *	1.00 (0.88–1.14)	0.965	0.95 (0.83–1.09)	0.496	

Abbreviations: CCI, Charlson Comorbidity Index; SBP, Systolic blood pressure; DBP, Diastolic blood pressure. * Conditional logistic regression analysis, Significance at *p* < 0.05. † Stratified model for age, sex, income, and region of residence. ‡ Model 1 was adjusted for SBP, DBP, fasting blood glucose, total cholesterol, and dyslipidemia history. § Model 2 was adjusted for model 1 plus obesity, smoking, alcohol consumption, CCI scores, benign paroxysmal vertigo history, vestibular neuronitis history, and other peripheral vertigo history.

**Table 3:** Crude and adjusted odd ratios (95% confidence interval) of date of lipophilic statin prescription (1 year) for Meniere’s disease with stratified subgroup according to age, sex, income, and region of residence.

Characteristics	Odds Ratios for Meniere’ Disease						*p* for Interaction
	Crude†	*p*-Value	Model 1 †‡	*p*-Value	Model 2 †§	*p*-Value	
Total participants (*n* = 38,670)							
Lipophilic statin prescription (1 year)	1.24 (1.14–1.36)	<0.001 *	0.95 (0.86–1.05)	0.308	0.90 (0.81–1.00)	0.058	
Age < 65 years old (*n* = 21,325)							0.941
Lipophilic statin prescription (1 year)	1.26 (1.09–1.45)	0.002 *	0.87 (0.74–1.02)	0.084	0.81 (0.68–0.97)	0.023 *	
Age ≥ 65 years old (*n* = 17,345)							
Lipophilic statin prescription (1 year)	1.23 (1.10–1.38)	<0.001 *	1.01 (0.89–1.16)	0.775	0.98 (0.85–1.12)	0.741	
Men (*n* = 13,760)							0.625
Lipophilic statin prescription (1 year)	1.24 (1.06–1.45)	0.007 *	0.93 (0.78–1.10)	0.406	0.87 (0.72–1.06)	0.161	
Women (*n* = 24,910)							
Lipophilic statin prescription (1 year)	1.24 (1.11–1.39)	<0.001 *	0.96 (0.85–1.09)	0.555	0.92 (0.80–1.05)	0.208	
Low income (*n* = 17,515)							0.760
Lipophilic statin prescription (1 year)	1.29 (1.12–1.47)	<0.001 *	0.92 (0.79–1.07)	0.297	0.87 (0.74–1.03)	0.106	
High income (*n* = 22,335)							
Lipophilic statin prescription (1 year)	1.21 (1.07–1.36)	0.002 *	0.97 (0.85–1.11)	0.647	0.92 (0.80–1.07)	0.274	
Urban residents (*n* = 16,335)							0.476
Lipophilic statin prescription (1 year)	1.17 (1.02–1.35)	0.022 *	0.90 (0.77–1.05)	0.177	0.85 (0.72–1.01)	0.070	
Rural residents (*n* = 22,335)							
Lipophilic statin prescription (1 year)	1.30 (1.15–1.46)	<0.001 *	0.98 (0.86–1.13)	0.864	0.93 (0.81–1.08)	0.336	

Abbreviations: CCI, Charlson Comorbidity Index; SBP, Systolic blood pressure; DBP, Diastolic blood pressure, * Conditional logistic regression analysis, Significance at *p* < 0.05, † Stratified model for age, sex, income, and region of residence. ‡ Model 1 was adjusted for SBP, DBP, fasting blood glucose, total cholesterol, and dyslipidemia history. § Model 2 was adjusted for model 1 plus obesity, smoking, alcohol consumption, CCI scores, benign paroxysmal vertigo history, vestibular neuronitis history, and other peripheral vertigo history.

**Table 4:** Crude and adjusted odd ratios (95% confidence interval) of date of hydrophilic statin prescription (1 year) for Meniere’s disease with stratified subgroup according to age, sex, income, and region of residence.

Characteristics	Odds Ratios for Meniere’ Disease						*p* for Interaction
	Crude †	*p*-Value	Model 1 †‡	*p*-Value	Model 2 †§	*p*-Value	
Total participants (*n* = 38,670)							
Hydrophilic statin prescription (1 year)	1.30 (1.08–1.55)	0.004 *	1.04 (0.86–1.25)	0.696	1.06 (0.77–1.47)	0.704	
Age < 65 years old (*n* = 21,325)							0.451
Hydrophilic statin prescription (1 year)	1.48 (1.11–1.96)	0.007 *	1.07 (0.80–1.44)	0.627	1.34 (0.80–1.33)	0.801	
Age ≥ 65 years old (*n* = 17,345)							
Hydrophilic statin prescription (1 year)	1.19 (0.94–1.51)	0.137	1.02 (0.80–1.29)	0.878	1.04 (0.80–1.34)	0.785	
Men (*n* = 13,760)							0.867
Hydrophilic statin prescription (1 year)	1.26 (0.94–1.70)	0.122	0.95 (0.70–1.29)	0.751	0.97 (0.69–1.36)	0.844	
Women (*n* = 24,910)							
Hydrophilic statin prescription (1 year)	1.38 (1.05–1.65)	0.016 *	1.11 (0.86–1.36)	0.498	1.17 (0.83–1.38)	0.590	
Low income (*n* = 17,515)							0.935
Hydrophilic statin prescription (1 year)	1.29 (1.98–1.71)	0.071	1.00 (0.75–1.33)	0.993	0.99 (0.72–1.36)	0.956	
High income (*n* = 22,335)							
Hydrophilic statin prescription (1 year)	1.30 (1.03–1.64)	0.027 *	1.07 (0.84–1.36)	0.574	1.08 (0.83–1.40)	0.550	
Urban residents (*n* = 16,335)							0.716
Hydrophilic statin prescription (1 year)	1.27 (0.97–1.66)	0.083	1.01 (0.77–1.33)	0.939	1.01 (0.74–1.37)	0.939	
Rural residents (*n* = 22,335)							
Hydrophilic statin prescription (1 year)	1.32 (1.04–1.68)	0.022 *	1.06 (0.83–1.36)	0.646	1.07 (0.82–1.40)	0.624	

Abbreviations: CCI, Charlson Comorbidity Index; SBP, Systolic blood pressure; DBP, Diastolic blood pressure. * Conditional logistic regression analysis, Significance at *p* < 0.05. † Stratified model for age, sex, income, and region of residence. ‡ Model 1 was adjusted for SBP, DBP, fasting blood glucose, total cholesterol, and dyslipidemia history. § Model 2 was adjusted for model 1 plus obesity, smoking, alcohol consumption, CCI scores, benign paroxysmal vertigo history, vestibular neuronitis history, and other peripheral vertigo history.

## Data Availability

Release of the data by the researcher is not allowed legally. All data are available from the database of Korea Centers for Disease Control and Prevention (kdca.go.kr). The Korea Centers for Disease Control and Prevention provides all of these data for any researcher who promises to follow the research ethics guidelines, with some cost. If you want to access the data of this article, you could download it from the website after promising to follow the research ethics guidelines.

## Supplementary Materials

The following are available online at https://www.mdpi.com/article/10.3390/ijerph18168692/s1, Table S1: Subgroup analysis of crude and adjusted odd ratios (95% confidence interval) of date of statin prescription (1 year) for Meniere’ disease by unstratified subgroup.
