# Supplementary material for: Association between Statin Use and Meniere’s Disease: Results from a National Health Screening Cohort
Source: Int J Environ Res Public Health. 2021 Aug 17;18(16):8692. doi: 10.3390/ijerph18168692 (PMC8392457; doi:10.3390/ijerph18168692)
Supplement: Supplementary file 1 [file ijerph-18-08692-s001.zip › ijerph-1306438-supplementary.pdf]

Table S1. Subgroup analysis of crude and adjusted odd ratios (95% confidence interval) of date of statin prescription (1 year) for Meniere′ disease by unstratified subgroup.

Characteristics	Odds ratios for Meniere′ Disease						P Value for Interaction
	Crude	P-Value	Model 1†	P-Value	Model 2‡	P-Value	
Underweight (n = 961)							
Statin prescription (1 year)	1.98 (0.94-4.13)	0.071	1.59 (0.70-3.64)	0.272	1.60 (0.63-4.04)	0.321	0.694
Normal weight (n = 13,644)							
Statin prescription (1 year)	1.18 (1.00-1.39)	0.045*	0.88 (0.73-1.06)	0.163	0.84 (0.68-1.03)	0.086	
Overweight (n = 10,505)							
Statin prescription (1 year)	1.34 (1.15-1.57)	<0.001*	1.00 (0.84-1.20)	0.977	0.96 (0.79-1.16)	0.651	
Obese (n = 13,560)							
Statin prescription (1 year)	1.21 (1.07-1.36)	0.003*	0.96 (0.83-1.10)	0.538	0.94 (0.81-1.09)	0.417	
Non-smoker (n = 30,609)							
Statin prescription (1 year)	1.29 (1.18-1.42)	<0.001*	0.99 (0.89-1.10)	0.857	0.86 (0.85-1.07)	0.457	0.298
Past smoker and current smoker (n = 8,061)							
Statin prescription (1 year)	1.17 (0.97-1.41)	0.106	0.86 (0.70-1.07)	0.179	0.78 (0.62-0.99)	0.043*	
Alcohol consumption < 1 time a week (n = 28,127)							
Statin prescription (1 year)	1.27 (1.15-1.40)	<0.001*	0.97 (0.86-1.08)	0.537	0.91 (0.81-1.03)	0.125	0.324
Alcohol consumption ≥ 1 time a week (n = 10,543)							
Statin prescription (1 year)	1.26 (1.08-1.48)	0.004*	0.95 (0.79-1.14)	0.582	0.95 (0.78-1.16)	0.602	
Systolic blood pressure < 140 mmHg and diastolic blood pressure < 90 mmHg (n = 29,010)							
Statin prescription (1 year)	1.28 (1.16-1.40)	<0.001*	0.94 (0.84-1.05)	0.250	0.91 (0.81-1.03)	0.124	0.651
Systolic blood pressure ≥ 140 mmHg or diastolic blood pressure ≥ 90 mmHg (n = 9,660)							
Statin prescription (1 year)	1.22 (1.04-1.43)	0.014*	1.03 (0.85-1.23)	0.789	0.95 (0.78-1.16)	0.623	
Fasting blood glucose < 100 mg/dL (n =24,311)							
Statin prescription (1 year)	1.32 (1.18-1.48)	<0.001*	0.95 (0.83-1.08)	0.452	0.91 (0.79-1.05)	0.182	0.907
Fasting blood glucose ≥ 100 mg/dL (n =14,359)							
Statin prescription (1 year)	1.22 (1.08-1.37)	0.001*	0.98 (0.85-1.12)	0.757	0.94 (0.81-1.09)	0.434	
Total cholesterol < 200 mg/dL (n = 19,973)							
Statin prescription (1 year)	1.21 (1.09-1.35)	<0.001*	0.90 (0.79-1.02)	0.104	0.88 (0.77-1.01)	0.076	0.390
Total cholesterol ≥ 200 mg/dL (n = 18,697)							
Statin prescription (1 year)	1.35 (1.18-1.53)	<0.001*	1.06 (0.92-1.23)	0.415	0.98 (0.84-1.15)	0.829	
CCI scores = 0 (n = 25,942)							
Statin prescription (1 year)	1.31 (1.17-1.46)	<0.001*	0.94 (0.83-1.07)	0.360	0.89 (0.78-1.03)	0.114	0.859
CCI scores = 1 (n =5,907)							
Statin prescription (1 year)	1.13 (0.95-1.35)	0.180	0.97 (0.79-1.19)	0.773	0.96 (0.77-1.20)	0.741	
CCI scores ≥ 2 (n = 6,821)							
Statin prescription (1 year)	1.17 (0.99-1.38)	0.068	0.95 (0.79-1.15)	0.622	0.93 (0.76-1.15)	0.512	

Non-dyslipidemia (n = 26,384)							
Statin prescription (1 year)	1.22 (0.97-1.52)	0.085	1.25 (1.00-1.53)	0.050	1.12 (0.87-1.43)	0.379	0.051
Dyslipidemia (n = 12,286)							
Statin prescription (1 year)	0.91 (0.82-1.01)	0.082	0.92 (0.83-1.02)	0.130	0.90 (0.81-1.01)	0.072	
Non-benign paroxysmal vertigo (n = 35,732)							
Statin prescription (1 year)	1.21 (1.10-1.32)	<0.001*	0.93 (0.83-1.03)	0.168	0.94 (0.84-1.05)	0.279	<0.001*
Benign paroxysmal vertigo (n = 2,938)							
Statin prescription (1 year)	0.86 (0.69-1.07)	0.172	0.92 (0.72-1.18)	0.522	0.90 (0.69-1.16)	0.397	
Non-vestibular neuronitis (n = 37,876)							
Statin prescription (1 year)	1.28 (1.17-1.39)	<0.001*	0.97 (0.88-1.07)	0.600	0.92 (0.83-1.02)	0.103	0.079
Vestibular neuronitis (n = 794)							
Statin prescription (1 year)	0.80 (0.50-1.26)	0.328	0.88 (0.53-1.48)	0.639	0.91 (0.56-1.60)	0.831	
Non-other peripheral vertigo (n = 36,612)							
Statin prescription (1 year)	1.27 (1.16-1.39)	<0.001*	0.98 (0.88-1.08)	0.630	0.96 (0.86-1.07)	0.439	<0.001*
Other peripheral vertigo (n = 2,058)							
Statin prescription (1 year)	0.82 (0.63-1.06)	0.133	0.80 (0.60-1.07)	0.136	0.74 (0.54-1.00)	0.048*	

CCI, Charlson Comorbidity Index; SBP, Systolic blood pressure; DBP, Diastolic blood pressure, * Unconditional logistic regression analysis, Significance at P < 0.05, † Model 1 was adjusted for age, sex, income, region of residence, SBP, DBP, fasting blood glucose, total cholesterol, and dyslipidemia history. ‡ Model 2 was adjusted for model 1 plus obesity, smoking, alcohol consumption, CCI scores, benign paroxysmal vertigo history, vestibular neuronitis history, and other peripheral vertigo history.
